# Understanding Cybersickness and Presence in Seated VR: A Foundation for Exploring Therapeutic Applications of Immersive Virtual Environments

**DOI:** 10.3390/jcm14082718

**Published:** 2025-04-15

**Authors:** Witold Pawełczyk, Dorota Olejarz, Zofia Gaweł, Magdalena Merta, Aleksandra Nowakowska, Magdalena Nowak, Anna Rutkowska, Ladislav Batalik, Sebastian Rutkowski

**Affiliations:** 1 Faculty of Physical Education and Physiotherapy, Opole University of Technology, 45-758 Opole, Poland; d690@student.po.edu.pl (A.N.); mag.nowak@student.po.edu.pl (M.N.); a.rutkowska@po.edu.pl (A.R.); 2 Descartes’ Error Student Research Association, Department of Physical Education and Physiotherapy, Opole University of Technology, 45-758 Opole, Poland; student.d.ol@po.edu.pl (D.O.); zofia.gawel@student.po.edu.pl (Z.G.); 3 Insitute of Health Sciences, University of Opole, 45-040 Opole, Poland; magdalena.merta@uni.opole.pl; 4 Department of Rehabilitation, University Hospital Brno, 62500 Brno, Czech Republic; batalik.ladislav@fnbrno.cz; 5 Department of Public Health, Faculty of Medicine, Masaryk University, 62500 Brno, Czech Republic

**Keywords:** VR, immersion, virtual walk, cybersickness, emotional response, spatial presence

## Abstract

**Background/Objectives:** To assess the spatial presence and impact of an immersive virtual reality (VR) walk on symptoms of cybersickness, emotions, and participant engagement, with the aim of providing insights applicable to future therapeutic VR interventions for individuals with limited mobility. **Methods:** The experiment involved 30 healthy individuals who used VR headsets while seated on chairs to experience a 360° virtual tour of the Venice Canals in Los Angeles. The effect of immersion was evaluated using the Virtual Reality Sickness Questionnaire (VRSQ) to measure cybersickness symptoms, the International Positive and Negative Affect Schedule-Short Form (I-PANAS-SF) to assess emotions, the Spatial Presence Experience Scale (SPES) to evaluate spatial presence, and the Flow State Scale (FSS) to quantify the flow state. **Results:** The results indicated that the virtual walk elicited both positive and negative reactions. The increase in eye strain (+0.66), general discomfort (+0.6), and headache (+0.43) was achieved in the VRSQ scale. Despite experiencing nausea and oculomotor symptoms, participants reported a high level of flow (range of scale items from 3.47 to 3.70), suggesting a beneficial impact of immersion on their well-being. Furthermore, the analysis of the I-PANAS-SF results revealed a predominance of positive emotions, indicating a favorable perception of the experience. However, the SPES scores exhibited variability in the perception of spatial presence (mean spatial presence score 3.74, SD 2.06), likely influenced by the characteristics of the visual material used. **Conclusions:** Overall, the immersive VR walk, despite the potential risk of cybersickness symptoms, as a seated passive exploration still promoted feelings of satisfaction and fulfillment, allowing the participants to actively engage with the virtual environment. These findings suggest that seated VR experiences hold promise as a tool for promoting well-being, but further research is needed to address cybersickness and optimize VR content for therapeutic use in populations with limited mobility.

## 1. Introduction

Sherman defines virtual reality (VR) as a computer-generated environment in which the user’s position and movement are tracked, while feedback for one or more senses is enhanced or entirely replaced by simulation. This process leads to the emergence of a sense of psychological immersion or physical presence in the digital world [1]. With the continuous advancement in technology, VR has found widespread applications across various fields, including medicine, education, rehabilitation, and the entertainment industry. The history of VR technology dates back to 1962, when Ivan Sutherland developed a device known as The Sword of Damocles, considered the first prototype of a VR system [2]. Initially, immersive technologies were primarily developed for military purposes, such as training soldiers, designing military equipment and vehicles, and preparing astronauts for demanding space missions [3,4]. In the following decades, VR gained significance in the civilian sector, leading to the development of advanced simulation systems used in education, therapy, and medical diagnostics. Currently, commercially available VR headsets are becoming increasingly widespread, and their popularity continues to grow. It is estimated that the number of VR users is around 66.8 million, and the market for this technology is expanding dynamically [5]. Modern VR systems focus on enhancing interaction with the virtual environment by increasing the realism of sensory experiences and improving ergonomics and user comfort. Research on VR encompasses both technological and psychological aspects, with a particular emphasis on its impact on perception, emotions, and cognitive processes.

Immersion is a key aspect of VR technology, defining the degree of user engagement in interaction with the digital environment. It can be defined as the subjective feeling of being present in a reality different from the one that physically surrounds the user [6]. The level of immersion depends on the technology used in a given VR system and the quality of its implementation, which includes both hardware and software aspects [7,8,9]. A high level of immersion allows the user to become fully absorbed in the virtual world, perceiving it as real. Achieving this effect is possible through the use of advanced simulation technologies that engage multiple senses, particularly vision and hearing, while minimizing the influence of stimuli from the real environment [10,11,12,13]. The degree of immersion affects the user experience, making it applicable in various fields such as training, therapy, rehabilitation, and entertainment. Depending on the level of immersion in the virtual world, VR can be classified into three categories: non-immersive, semi-immersive, and fully immersive [14,15]. Non-immersive VR allows for limited interaction with the digital environment, whereas semi-immersive systems provide greater sensory engagement while still keeping the user aware of the real surroundings. Fully immersive VR enables complete immersion in the virtual world and real-time interaction, leading to a maximum sense of presence and psychological engagement in the simulated reality [16].

VR can significantly influence the user’s mental state, affecting the phenomenon known as “flow”—an optimal state of mind characterized by high motivation and full engagement in a given activity. This state occurs when individuals engage in challenging yet intrinsically motivating tasks [17,18]. The experience of flow plays a crucial role, as it not only enhances the subjective feeling of enjoyment at a given moment but also contributes to increased self-confidence and supports the development of personal competencies [19]. This phenomenon can manifest in various aspects of daily life regardless of the field of activity [17]. Since its initial characterization, this phenomenon has been the subject of numerous scientific studies due to its significance for various aspects of human functioning [20]. In particular, it has been proven that the state of flow enhances creativity, improves the process of learning and skill acquisition, and maximizes performance in sports [21,22].

Research on the experience of spatial presence in the context of immersive walking is well established and provides valuable insights, particularly in areas such as virtual tourism [23]. The popularization of VR has contributed to the development of virtual tourism, allowing users to explore any location on Earth without the need to leave their homes [24]. It is a virtual experience that provides users with a full sense of presence in VR through advanced technologies and devices such as VR headsets, sensory gloves, and motion platforms [25]. The user can move and interact with the virtual world, enabling full engagement and a more realistic experience than simple virtual sightseeing. In the tourism industry, VR is gradually finding its applications, allowing companies to showcase destinations offered in their travel packages. Google provides the Google Earth VR service, which enables users to explore any location captured by the company. A similar tool is Realities, an application that allows users to visit various locations. Although its database is not as extensive as Google’s, it utilizes a more advanced method of generating virtual environments—photogrammetry, which enhances realism [26]. One of the key benefits of virtual tourism is the ability to explore an interesting location without the need for physical presence, especially if the chosen destination turns out to be less engaging than expected. Another advantage is the absence of crowds in the visited location, allowing users to explore museums or other attractions without waiting in long queues and with nearly unrestricted freedom of movement and time constraints. Moreover, for disabled patients, particularly those with severe physical limitations, engaging with the external world can be challenging. VR offers a promising avenue for these individuals to experience immersive environments and potentially improve their psychological well-being [27].

Despite its numerous advantages and wide range of potential applications, the use of VR can also lead to side effects. One of the most frequently reported negative effects is cybersickness, a syndrome characterized by discomfort, apathy, and sensory disturbances. This condition is often classified as a form of motion sickness induced by visual stimuli, meaning its mechanisms may be similar to kinetosis caused by visually perceived motion [28]. The most common symptoms of cybersickness include nausea, disorientation, and general discomfort, which significantly affect users’ ability to engage with VR systems for extended periods [29]. The mechanism behind these symptoms is explained by the sensory conflict theory, which suggests that a discrepancy between the information received by the visual system and the lack of corresponding stimulation of the vestibular system leads to physiological reactions associated with balance disturbances and altered spatial perception [30,31]. It is estimated that up to 80% of VR users may experience symptoms of cybersickness after just 10 min of exposure to a virtual environment [32]. The Simulation Sickness Questionnaire (SSQ) is the most commonly used tool for assessing the severity of cybersickness symptoms; however, it has been criticized for methodological limitations [33]. As a result, alternative questionnaires have been developed, including the Cybersickness Questionnaire (CSQ), VR Sickness Questionnaire (VRSQ), and The Positive and Negative Affect Schedule (PANAS), which aim to provide a more precise evaluation of VR’s impact on user well-being [33,34]. The impact of spatial presence experience on students under the age of 25 remains an underexplored area. This study aims to fill this research gap by analyzing the mechanisms of spatial presence perception, its detection in the context of VR, and the evaluation of emotions associated with participation in VR.

## 2. Materials and Methods

The study involved 30 participants, including 14 men (47% of the sample) and 16 women. The average age of the participants was 23 years, with an age range between 20 and 25 years. Among all the participants, 13 individuals (43%) reported no prior experience with using VR headsets. The study adhered to the Declaration of Helsinki, ethical approval was obtained from the Bioethics Committee of the Opole Chamber of Physicians on the basis of Resolution No. 243 of 6 April 2017.

The visual materials used in the study were displayed via the Meta Quest 2 headset (Meta Platforms, Inc., Menlo Park, CA, USA) and were appropriately adapted for the VR mode. During the experiment, the participants watched recordings available on the YouTube platform, depicting a virtual walk through the Venice Canals in Los Angeles. The Venice Canals location was specifically selected due to its visually engaging scenery, atmosphere, and popularity as a representative virtual tourism destination.

The duration of the recorded content was approximately 15 min, and the footage was captured using a 360° camera, allowing for full immersion in the virtual environment.

**Figure 1:**
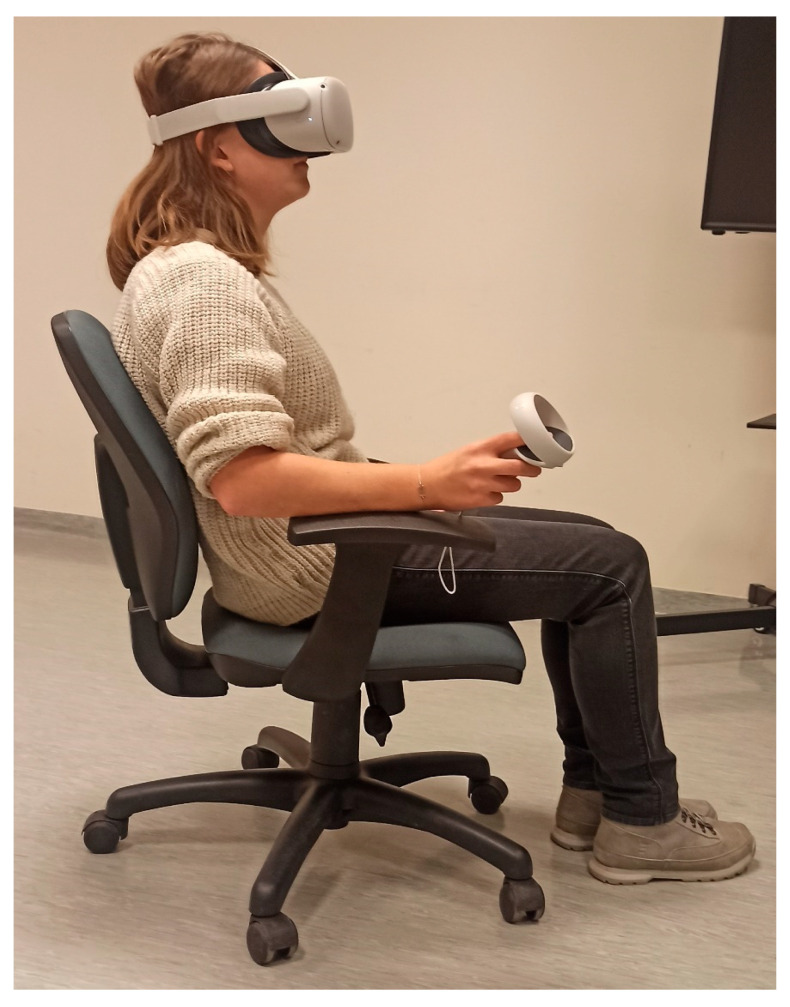
Experimental setup: participant using a VR headset.

Thanks to the use of this technology and VR headsets, the participants were able to freely explore the camera’s field of view by rotating their heads in any direction and observing the environment from different perspectives. Additionally, the recording included audio effects, such as the sound of flowing water and birdsong, which enhanced the realism of the experience and strengthened the sense of presence in the simulated environment. During the experiment, the participants sat on a rotating chair, which facilitated their interaction with the virtual environment and allowed for unrestricted head and body movement. The participants were instructed to rotate freely yet gently, according to their comfort, without excessive or rapid movements, to minimize uncontrolled rotations that might influence cybersickness symptoms. The testing room was kept completely silent to minimize external stimuli and maximize the participants’ focus on the VR experience (Figure 1).

The participants were asked to complete questionnaires assessing their subjective experiences. The VRSQ and I-PANAS-SF questionnaires were completed both before and after the experiment, whereas the FSS and SPES were administered only after the experiment. Among the measurement tools used was the Virtual Reality Sickness Questionnaire (VRSQ). This questionnaire consists of 16 questions that evaluate symptoms of cybersickness, including the severity of nausea, oculomotor disturbances, and disorientation. The assessment was conducted using a five-point scale (0–4) [29]. Another measurement tool used was the International Positive and Negative Affect Schedule-Short Form (I-PANAS-SF). This ten-item questionnaire assessed the positive and negative emotions experienced by the participants after exposure to the virtual environment. To measure the flow experience, the Flow State Scale (FSS) was applied. This tool consists of 9 questions and is used to evaluate the state of full engagement, concentration, and internal harmony while performing an activity and is adapted to the specific nature of interactions within VR. The final research tool was the Spatial Presence Experience Scale (SPES), an eight-item questionnaire with a 6-point scale designed to assess the sense of spatial presence in VR, allowing for the determination of the extent to which the participants experienced immersion and physical presence in the simulated environment. The application of these tools enabled a comprehensive analysis of the impact of immersive VR experiences on the participants and an assessment of their potential psychophysiological effects.

## 3. Statistical Analysis

The data obtained in the study were recorded in Microsoft Excel and subsequently analyzed using the STATISTICA 13 and JASP 0.18.1 software. To verify the statistical significance of the differences in the results, a non-parametric test for dependent samples—the Wilcoxon signed-rank test—was applied. A significance level of α = 0.05 was adopted for statistical analysis [35]. The sample size calculation for this study was informed by existing research in the field of Virtual Reality Exposure Therapy, which generally reports an average effect size of approximately 0.5 for anxiety disorders. This estimate was used to ensure adequate statistical power for detecting significant effects in our investigation; it was determined that 28 participants should be enrolled [36]. The G*Power 3.1.9 software was used to calculate the sample size. The calculation was based on the Wilcoxon signed-ranks test, and the type I error rate was set at 5% (α = 0.05), the effect size of the main outcomes was 0.5, and the type II error rate gave 80% power.

## 4. Results

**Figure 2:**
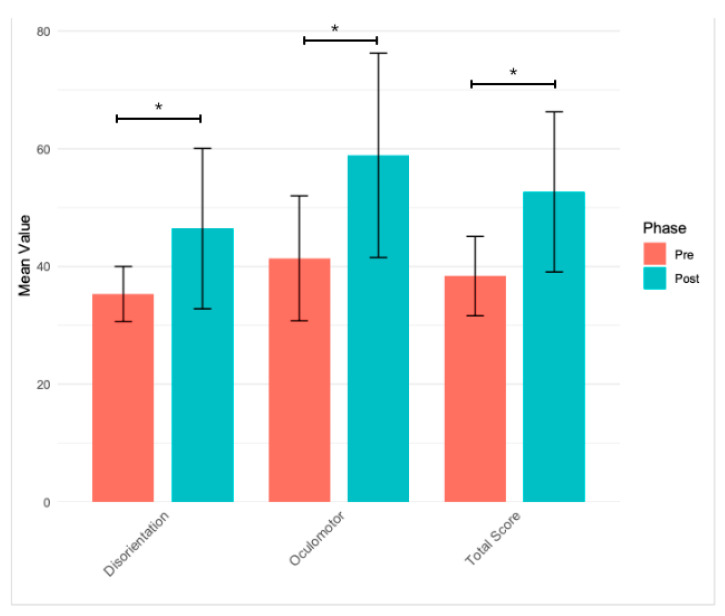
Changes in cybersickness indicators pre-and post-experiment (VRSQ scores); * *p* < 0.05.

The analysis of the VRSQ results indicated that the participants experienced an increase in cybersickness symptoms following exposure to the VR environment. The most pronounced increase was observed in eye strain (+0.66), general discomfort (+0.60), and headache (+0.43). After completing the VR projection, the participants more frequently reported eye fatigue, physical discomfort, and mild headaches, suggesting that the immersive experience had a noticeable impact on their well-being. Smaller changes were noted in difficulty concentrating (+0.34) and fatigue (+0.17). (Table 1, Figure 2).

**Figure 3:**
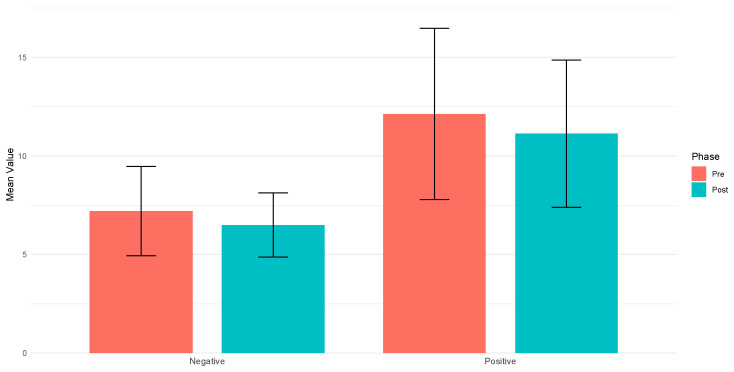
Positive and negative affect scores before and after VR exposure (I-PANAS-SF).

To assess changes in the level of experienced emotions, a comparative analysis of the PANAS scale results before and after the intervention was conducted. This scale measures two dimensions of affect: positive and negative emotions. The analysis revealed that the mean score for positive emotions before the intervention was 12.2, which decreased to 11.1 after the intervention. In the case of negative emotions, the mean score was 7.2 before the intervention and decreased to 6.7 afterward. The Wilcoxon signed-rank test did not indicate statistically significant differences in the level of emotions. The *p*-value was 0.0971 for positive emotions and 0.1511 for negative emotions (Table 2, Figure 3).

The analysis of the Spatial Presence Experience Scale results indicated that the participants experienced a moderate level of spatial presence in the virtual environment. The mean spatial presence score was 3.74 (SD = 2.06), which shows that most participants felt a sense of presence in the virtual environment, although these perceptions varied. The highest mean scores were observed for physical presence in the VR environment (M = 3.33, SD = 1.79) and the sense of participation in the presentation’s action (M = 3.28, SD = 1.83). The lowest scores were recorded for the perception of the ability to interact with objects in the VR environment (M = 2.61, SD = 2.44) (Table 3).

The analysis of the Flow State Scale results indicates that the participants experienced a moderate level of flow during the VR experiment. The mean scores for all the scale items ranged from 3.47 to 3.70, showing that most participants generally agreed with the statements describing the flow state. The highest flow levels were reported in terms of feeling competent to meet situational demands (M = 3.70, SD = 1.12) and acting spontaneously and automatically, without the need for conscious thought (M = 3.63, SD = 1.27). The lowest scores were observed for a strong awareness of purpose during the task (M = 3.47, SD = 1.17) and self-evaluation of performance effectiveness (M = 3.47, SD = 1.01). The standard deviation values for most items ranged from 1.01 to 1.27 (Table 4).

## 5. Discussion

The aim of this study was to analyze the impact of virtual tourism technology on users. The findings confirm the positive effects of the virtual walk experience on the participants’ perceptions. The participants demonstrated a higher level of positive emotions compared to negative emotions, as assessed during the study. These results suggest that virtual walking experiences influence users’ emotions and overall perception. The analysis of the questionnaires SPES, I-PANAS-SF, and FSS allow for the conclusion that passive immersive VR exploration, as experienced in a seated position to better control experimental conditions, can elicit positive emotional responses and a sense of flow, even with the potential for cybersickness symptoms.

Regarding the VRSQ, the study identified an impact of the virtual walk on symptoms related to nausea. This observation is consistent with the findings from previous studies, which also report that immersive VR experiences can induce symptoms of cybersickness, particularly nausea [37]. This indicates that the participants experienced greater discomfort after the VR session compared to their pre-exposure state, with symptoms such as general discomfort, fatigue, headaches, and eye strain. These findings align with previous studies, which have also reported an increase in cybersickness symptoms following immersive VR experiences [38]. Additionally, recent research highlights the importance of camera stabilization in 360° videos as a critical factor for reducing cybersickness symptoms, suggesting that stabilizing visual input could enhance the quality and comfort of passive VR experiences [39]. Practical implications from these findings include implementing effective stabilization techniques to minimize visual discomfort and improve user experience in similar VR applications.

A different pattern of symptom dominance was observed in Palmisano’s study on the effects of VR gaming on cybersickness symptoms in individuals aged 18–30 years. In that study, the most frequently reported symptom was disorientation following the VR experience, whereas nausea and oculomotor discomfort were reported less frequently [40]. Analyzing the impact of the virtual walk on oculomotor symptoms, it can be concluded that the participants’ scores worsened compared to their pre-exposure values, a finding that is also supported by previous studies. Lee demonstrated that oculomotor symptoms and dizziness can appear after just five minutes of VR headset use. Additionally, the study showed that these symptoms are significantly more pronounced after 20 min of exposure compared to just five minutes [37]. In the present study, the projection duration was 15 min, without any break, which was sufficient for the onset of significant symptoms, as assessed using the VRSQ. The occurrence of VR-induced dizziness should be considered when evaluating user safety. The seated position used in this study appears to minimize the risk of falls associated with balance disturbances. Palmisano’s findings suggest that cybersickness symptoms may decrease over repeated VR sessions, with the greatest intensity occurring during the first exposure. This indicates a potential for the body to gradually adapt to the VR environment over time [40]. The VRSQ proved to be a more effective tool in assessing the impact of VR technology, as it was completed both before and after the experiment, allowing for a direct comparison of symptoms.

Similar results were observed in a study conducted by Kim et al., which included 33 participants divided into three groups. The first group consisted of 11 individuals aged 28 ± 7 years. The second group included 11 participants aged 66 ± 3 years. The third group also consisted of 11 participants, all of whom suffered from Parkinson’s disease, with an average age of 65 ± 7 years. The study aimed to examine the effects of a virtual walk on participants, with a particular focus on individuals with Parkinson’s disease. It assessed the impact of the virtual environment on cybersickness symptoms and measured spatial presence levels. In several cases, even though participants verbally reported no symptoms, their scores on the cybersickness questionnaire suggested a higher-than-average level of symptoms compared to the other groups, indicating a discrepancy between subjective reports and objective assessments [41]. The results of the study, comparing pre- and post-experiment scores in the age group of 28 ± 7 years, indicated a negative impact of the experiment, similar in magnitude to the findings of the present study. Similarly, Lee et al. examined symptoms of cybersickness and their relationship with different forms of interaction in a virtual environment, including a game controller, a motion-responsive interface, and a walking simulation. The experiment involved 20 participants aged between 20 and 35 years. In this study, each participant underwent a virtual walking session using each type of controller, with cybersickness symptoms measured after each session using a standardized questionnaire. However, the study did not provide baseline data collected before the experiment, making it difficult to accurately assess the direct impact of VR exposure on cybersickness symptoms. The results of the cybersickness questionnaire showed higher scores compared to the other participants in the study, particularly in the domain of disorientation, which was the most affected symptom category [42]. The conducted study also aimed to assess the level of immersion and spatial presence, which, depending on the type of controller used, reached moderate to high scores. These results were higher than those obtained in the present study, which can be attributed directly to the nature of the experimental setup. The ability to interact with the virtual environment likely contributed to a greater sense of presence compared to the passive viewing experience used in the current research.

The results of the Spatial Presence Experience Scale indicate a moderate level of spatial presence reported by participants. The lowest scores were observed for questions related to interaction with the virtual environment, which can be attributed to the study’s methodology, where no interactive elements were included. However, questions focusing on presence and the experience of being within the virtual space yielded higher spatial presence scores. Elmezeny et al. suggested in their studies that 360-degree VR experiences can significantly enhance the sense of presence, as visual cues reach the user directly, creating a stronger perceptual connection to the virtual environment [43]. The variability in responses in the author’s own study highlights significant individual differences in the experience of spatial presence. The variability in participants’ experiences of spatial presence and cybersickness symptoms may be partially explained by individual differences in autonomic nervous system responses. Similar findings have been reported in studies investigating ANS reactivity in nociplastic pain conditions, where heightened autonomic responses were observed in specific pathological contexts [44,45]. Some participants exhibited a strong sense of immersion, while others did not feel a substantial transfer of their presence into the virtual environment. This may suggest the influence of individual factors, such as prior VR experience, level of engagement with the content, or personal susceptibility to immersion effects. For the Flow State Scale (FSS) questionnaire, the results suggest that the participants experienced a moderate level of flow across all the measured components, as indicated by mean scores around 3.5. The high flow levels reported in terms of feeling competent to meet situational demands and acting spontaneously and automatically, without the need for conscious thought, suggest that the participants generally felt comfortable and confident in the VR environment, which may have contributed to their engagement and fluidity of action. The low scores observed for a strong awareness of purpose during the task and self-evaluation of performance effectiveness suggest that while the participants were engaged in the VR experience, they did not always have a clear awareness of the objective of their actions within the simulation. The standard deviation values for most items ranged from 1.01 to 1.27, indicating some degree of individual variability in the flow experience. The participants differed in their levels of concentration, sense of control, and satisfaction derived from the VR experience, which could be influenced by individual personality traits, prior VR experience, or the level of immersion in the presented content.

The I-PANAS-SF analysis required dividing the results into two categories: positive and negative emotions. The key aspect of this scale is the dominance of one emotional category over the other—for example, when the total score for negative emotions exceeds the total score for positive emotions. In this study, the mean score for positive emotions was 11.1 ± 3.67, while the mean score for negative emotions was 6.5 ± 1.61. The higher score for positive emotions suggests that the VR experience was generally well received and had a positive influence on participants’ emotional states.

However, the findings from Le’s study indicate that it is difficult to determine the direct impact of the VR experience on the participants’ emotions solely based on the post-experiment questionnaire results, as I-PANAS-SF was administered only after the intervention without a baseline measurement [46]. Based on the results obtained regarding spatial presence and flow experience, it can be concluded that the participants experienced these phenomena at a moderate level. This outcome was likely influenced by the study design, which involved a seated position and passive video playback, limiting the participants’ ability to interact with the virtual environment.

One limitation of the study was the sample size of 30 participants, which did not allow for precise validation of the research hypotheses. Additionally, all the participants were young and healthy, which suggests that further research should be conducted on different population groups to generalize the findings. Future studies could investigate the adaptation effects of repeated VR exposures, compare the outcomes of passive versus interactive VR experiences, and explore cybersickness in populations with limited mobility, such as hospitalized or elderly individuals. Future research should focus on adapting VR content and hardware specifically for bedridden patients. This may include optimizing the viewing experience for a lying position, developing interfaces that can be controlled with minimal movement (e.g., eye tracking), and investigating methods to minimize cybersickness in this population. Additionally, studies should examine the long-term effects of VR on mood, cognitive function, and social interaction in bedridden patients.

A virtual tour has the potential to be used as a therapeutic tool in the rehabilitation of hospitalized patients. This is particularly relevant for bedridden individuals, as demonstrated in a study by Gerber et al., where the use of VR technology was found to have a beneficial impact on users, helping them reduce stress and achieve relaxation [47]. Knibbe et al. demonstrated similar effects of VR on participants in their study. Additionally, due to the nature of the materials used, an increase in physical activity was observed. However, it is important to note that the lying position of the participants contributed to an increase in cybersickness symptoms, as a sensory conflict emerged: in the virtual environment, the user appeared to be standing, while in reality, they remained lying down. However, after some time, most participants reported that they had “forgotten they were lying down,” indicating a shift in their perception of body position within the virtual environment [48]. Practically, the presence of cybersickness may limit users’ willingness to engage repeatedly with passive VR environments, thus emphasizing the need for technical and methodological improvements—such as optimized camera stabilization, gradual exposure protocols, or repeated sessions to promote user adaptation and reduce negative symptoms. Therefore, it should be used as a non-pharmacological intervention to provide a sense of “travel” or exploration, potentially combating feelings of isolation and depression often experienced by long-term bedridden individuals.

## 6. Conclusions

The analysis of the results showed that the participants experienced a flow state, which is a positive psychophysiological condition characterized by deep engagement and immersion in the activity. The I-PANAS-SF questionnaire results indicate a positive reception of the VR projection by the participants, suggesting that the virtual walk had a beneficial impact on their well-being and emotional state. These findings support the potential of VR as a tool for inducing positive emotional states. However, in 70% of the participants, differences were observed between the pre- and post-exposure values in the nausea category and oculomotor symptoms group. The participants reported increased discomfort, including general unease, fatigue, headaches, and eye strain. Disorientation was among the most strongly perceived symptoms, with its intensity varying based on the individual predispositions of the participants. Despite these challenges, the positive emotional responses and sense of flow experienced by the participants suggest that VR is noteworthy tool.

## Figures and Tables

**Table 1:** Changes in cybersickness symptoms before and after VR exposure.

Indicator	Pre Mean (SD)	Post Mean (SD)	Delta (Post-Pre)	Wilcoxon *p*-Value
General Discomfort	1.17 (0.38)	1.77 (0.63)	0.6	0.0001
Fatigue	1.53 (0.63)	1.7 (0.7)	0.17	0.1967
Eye Strain	1.37 (0.56)	2.03 (0.81)	0.66	0.0002
Difficulty Concentrating	1.23 (0.43)	1.57 (0.68)	0.34	0.0124
Headache	1.07 (0.25)	1.5 (0.78)	0.43	0.0059
Mental Fog	1.17 (0.38)	1.47 (0.68)	0.3	0.0293
Blurred Vision	1.03 (0.18)	1.43 (0.63)	0.4	0.003
Feeling of Swaying with Eyes Closed	1.03 (0.18)	1.3 (0.53)	0.27	0.0114
Dizziness	1.0 (0.0)	1.27 (0.52)	0.27	0.0114
Oculomotor Symptoms	41.39 (10.61)	58.89 (17.36)	17.5	0.0001
Disorientation Symptoms	35.33 (4.68)	46.44 (13.62)	11.11	0.0001
Total Score	38.36 (6.74)	52.67 (13.6)	14.31	<0.0001

**Table 2:** Comparative analysis of positive and negative affects before and after VR experience (I-PANAS-SF Results).

	Positive	Negative
**Mean (SD) Pre**	12.13 (4.34)	7.2 (2.27)
**Mean (SD) Post**	11.13 (3.73)	6.5 (1.63)
**Delta (Post-Pre)**	−1.0	−0.7
**Wilcoxon *p*-value**	0.097	0.151

**Table 3:** Flow experience during virtual walk (SPES results).

Survey Item	Mean Score	Standard Deviation
I felt like I was actually there in the environment of the presentation.	3.74	2.06
It seemed as though I actually took part in the action of the presentation.	3.28	1.83
I had the impression that I was in the same place as the characters and/or objects in the presentation.	3.25	1.75
I felt as though I was physically present in the environment of the presentation.	3.33	1.79
I felt as though I could interact with the characters and/or objects in the presentation.	2.61	2.44
I had the impression that I could act in the environment of the presentation.	2.83	2.13
I had the impression that I could move around among the characters and/or objects in the presentation.	3.09	1.88
It seemed to me as though I could do whatever I wanted in the environment of the presentation.	2.68	2.33

**Table 4:** Flow experience during virtual walk (FSS results).

Survey Item	Mean Score	Standard Deviation
I felt competent enough to meet the demands of the situation.	3.7	1.12
I acted spontaneously and automatically, without having to think.	3.63	1.27
I had a strong sense of what I wanted to do.	3.47	1.17
I had a good idea of how well I was doing when engaged in the task/activity.	3.47	1.01
I was completely focused on the task at hand.	3.53	1.11
I felt a complete sense of control over what I was doing.	3.3	1.24
I was not worried about what others might think of me.	3.83	1.34
The way time passed seemed different than usual.	4.07	1.01
The experience was extremely satisfying for me.	3.73	1.14

## Data Availability

The data presented in this study are available upon request from the corresponding author.
